# Larval nutritional-stress and tolerance to extreme temperatures in the peach fruit fly, *Bactrocera zonata* (Diptera: Tephritidae)

**DOI:** 10.1080/19336934.2022.2157161

**Published:** 2022-12-28

**Authors:** M. Ben-Yosef, Y. Altman, E. Nemni-Lavi, N.T. Papadopoulos, D Nestel

**Affiliations:** a Department of Entomology, Institute of Plant Protection, Agricultural Research Organization, Rishon Letzion, Israel; b Laboratory of Entomology and Agricultural Zoology, Department of Agriculture Crop Production and Rural Environment, University of Thessaly, Volos, Greece

**Keywords:** *Bactrocera zonata*, thermal tolerance, t_max_, environmental stress, nutritional state, cold tolerance

## Abstract

Within the factors affecting insect tolerance to extreme environmental conditions, insect nutrition, particularly of immature stages, has received insufficient attention. In the present study, we address this gap by investigating the effects of larval nutrition on heat and cold tolerance of adult *Bactrocera zonata* – an invasive, polyphagous fruit fly pest. We manipulated the nutritional content in the larval diet by varying the amount of added yeast (2–10% by weight), while maintaining a constant sucrose content. Adults derived from the different larval diets were tested for their tolerance to extreme heat and cold stress. Restricting the amount of yeast reduced the efficacy of the larval diet (i.e. number of pupae produced per g of diet) as well as pupal and adult fresh weight, both being significantly lower for yeast-poor diets. Additionally, yeast restriction during the larval stage (2% yeast diet) significantly reduced the amount of protein but not lipid reserves of newly emerged males and females. Adults maintained after emergence on granulated sugar and water for 10 days were significantly more tolerant to extreme heat (i.e. knock-down time at 42 ^o^C) when reared as larvae on yeast-rich diets (8% and 10% yeast) compared to counterparts developing on a diet containing 2% yeast. Nevertheless, the composition of the larval diet did not significantly affect adult survival following acute cold stress (exposure to −3°C for 2 hrs.). These results are corroborated by previous findings on Drosophilid flies. Possible mechanisms leading to nutrition-based heat-tolerance in flies are discussed.

## Introduction

The capacity of an individual or a species to tolerate extreme environmental conditions is ultimately determined by a complex of inherited morphological, physiological, metabolic and behavioural traits [1]. Many of these are liable to modulation during ontogenesis through interaction with the environment, while others remain genetically predetermined [2]. The plasticity of such traits eventually determines the ability of an individual or a species to adapt to changes and withstand extreme conditions in their habitat [3].

The effects of dietary restriction and nutritional status on fitness and longevity have been extensively studied in insects, which serve as an excellent proxy to manipulate nutritional status and to explore consequent effects on metabolic regulation and genetic plasticity [e.g. 4]. Nevertheless, the association between nutrition and tolerance to thermal and other climatic stressors has been less explored [5,6], and in tephritid fruit flies such studies are largely limited to the adult stage. For instance, adult *Ceratitis capitata* and *C. rosa* were found to be less tolerant to extreme temperatures when starved [7,8]. Similarly, Colinet & Renault [9], found that by feeding on protein-rich diets during adulthood, *Drosophila melanogaster* better tolerate cold stress, possibly due to an increase in body mass and accumulation of metabolic reserves. Nevertheless, the effects of larval nutrition on adult tolerance to thermal stress are less studied [6,10].

Holometabolous insects may experience limitation in nutritional resources during their development, affecting their life history and fitness [4,10]. Herbivores, such as tephritid larvae, are often confined to feeding on nutritionally restricted diets, having low protein content. Understanding the effects of nutritional deficiencies during early stages of development on fitness and stress tolerance of the resulting adults could provide cues on the mechanisms used to cope with nutritional imbalances and promote our understanding of the evolutionary biology of species and their potential to expand their geographic range to new environments [4,6,10]. Such conclusions may be pertinent for accurately modelling the geographic distribution of pests, particularly in light of the changing climate [11,12]. Temperature and humidity are considered the most important environmental stressors affecting insects [8], and the ability of insects as ectothermic animals to endure in their habitat and expand into new geographic areas is directly associated with their capacity to adapt to temperature and humidity changes and tolerate extreme climatic events [e.g. 13].

The Peach Fruit Fly (*Bactrocera zonata*, PFF) is a polyphagous, invasive tephritid originating in tropical South-East Asia [14]. PFF has been progressively expanding its geographic range during the last decades, reaching areas of Northern Africa and the Middle-East [15]. As with other polyphagous tephritid fruit flies, their detection and colonization in new regions induces plant protection agencies to launch expensive eradication programmes to protect their fruit producing industries [16]. Moreover, their detection in fruit exporting regions may lead importing countries to establish restriction to the movement of produce from exporting countries [16]. In Israel, the PFF is frequently detected in urban and semi-urban regions of the Tel Aviv metropolitan area [17] and is currently considered as a major invasive species for Mediterranean and Continental Europe [15]. Restrictions, which include the utilization of the system approach [16], have been requested by European countries to the exports of citrus from Israel to the EU.

In an effort to understand and model the capacity of the fly to expand its geographic range, our group has recently embarked on characterizing the tolerance of the species to hydric stress [18] and thermal stress (unpublished data in preparation for publication). In this study, we address the effects of larval nutrition on the thermal tolerance of the PFF. More specifically, we examined the effects of yeast availability during the larval stage on life history traits and tolerance to thermal stress of the PFF.

## Material and methods

### Larval diet composition and experimental setup

*Bactrocera zonata* eggs were obtained from a laboratory colony maintained in the Israeli Plant Protection and Inspection Services quarantine laboratories. The colony has been routinely propagated on an artificial 8% brewer’s yeast larval diet (see Table 1 for other components in this diet). Every year a fresh colony is initiated from wild material [see 19 for details, and Y. Gazit personal communications]. The amount of yeast in the above artificial diet was modified to establish the experimental diets for the larval development assays. Specifically, diets containing 2%, 4%, 8% or 10% yeast (by weight), and a constant level of 12% sucrose were established. The amount of bulking agent (wheat bran) was adjusted according to the content of yeast added to the diet (for specific diet composition see Table 1). Petri dishes containing 20 g of each of the above diet regimes (i.e. experimental unit) seeded with approximately 600–800 eggs (ca. 30–40 eggs/g of diet) were incubated at 25°C under ambient humidity until the larvae had completed their development and left the diet to pupate. This is a relatively dense population of eggs and larvae per g of substrate. This approach allowed us to ‘augment’ the effects of larval diet nutrient-content on the fly’s physiology and biology, emphasizing differences on the measured biological parameters [see for example, 20]. A sand trey placed under each petri dish was used to collect the pupae, which were subsequently maintained at 25°C, counted, and weighed to compare the rearing efficiency, pupal weight and thermal stress resistance of adults of different treatment groups (see below). These experiments were replicated three times using eggs of different rearing cohorts. Each experiment consisted of five experimental units per treatment.

**Table 1.** Larval-diet composition per 100 g of diet.

	% yeast in diet			
Constituent(g | ml)	2	4	8	10
Wheat bran	32.8	30.8	26.8	24.8
Brewer’s yeast	2	4	8	10
Sucrose	12	12	12	12
Sodium benzoate	0.4	0.4	0.4	0.4
HCl	1.6	1.6	1.6	1.6
Water	51.2	51.2	51.2	51.2
Total Weight	100	100	100	100

### Larval performance assays

We determined the effect of diet composition on larval rearing efficiency by recording the number of pupae obtained for each g of diet used. Pupal weight was determined by individually weighing 20 randomly sampled pupae from each treatment group 5 days after pupation. A second sample of 20 pupae taken from each treatment was placed in a cage to evaluate adult emergence rate and flying ability following standard procedures [21]. The remaining pupae were allowed to develop, and the emerging adults were sorted by sex and maintained separately in cages containing granular sucrose and water for the next 10 days. Protein was not provided in order to avoid possible compensation for deficiencies accumulated during the larval stage [4]. By restricting protein, we expected to keep flies derived from all diets in a ‘reproductive waiting-mode’ [22], reducing the possibility of remediation of deficiencies carried-over from the larval stage and keeping nutritional shortages for further testing. By being in a reproductive waiting-mode, we also expected flies derived from different larval-diet treatments to keep metabolic regulation alike (i.e. metabolic ‘maintenance’). Males and females were subsequently tested for their resistance to extreme temperature conditions (see following section). At the day of emergence, we additionally sampled 5–15 individuals of each sex, from each treatment group in order to analyse their individual protein and lipid contents, following conventional extraction and quantification methods as previously described (see [23]). Briefly, soluble protein was extracted by homogenizing individual flies in Phosphate Buffered Saline, and colorimetrically quantified using the Bradford method. Lipids were extracted by homogenizing flies in 2% sodium sulphate solution and separated from polar substances with a 1:1 mix of chloroform and methanol. After evaporating the solvents, lipids were hydrolysed with sulphuric acid and colorimetrically quantified using the vanillin-phosphoric acid reagent. All of these assays were performed for each of the three performed replicates.

*Resistance to extreme temperature conditions*:

We tested the effect of larval diet on the tolerance of 10-day old, pre-weighed males and females to extreme high (42°C) and extreme low (−3°C) temperatures. Preliminary studies with *B. zonata* and *C. capitata* [24] were used to establish the working protocols. Exposure of adults to 0°C for 2 hr. did not show adult mortality (unpublished data); thus, we decided to use −3 ^o^C to investigate effects of larval nutrition. This temperature did affect survival, thus, allowing us to explore the effects of larval nutrition on cold tolerance. The chill temperature used provides information on the tolerance of the species to cold and to the possibilities of the species to tolerate climates bearing these temperatures during winter in regions with host prone to be ‘colonized’ by the species, like Continental Europe.

Exposure to high-temperature stress was conducted using a double jacketed chamber (‘organ-pipe’ system) connected to a circulating water source which was gradually heated (0.5°C per minute, starting from 25°C) to 42°C [see 25]. Insects were individually introduced to the chambers of the ‘organ-pipe’ system when the temperature was 25°C. When temperature reached 42°C we started to monitor time and registered the time needed for the fly to completely lose neuro-muscular coordination (i.e. knock-down) resulting from overheating [25]. Three replicates (representing different rearing cohorts) were conducted where flies of all treatment groups were assayed on the same day (overall, n = 9–10 flies of each sex for each diet, total n = 78 flies).

To determine the adult tolerance to acute cold stress, males and females were individually confined to 1.5 ml Eppendorf tubes and suspended in a pre-chilled water bath containing ethylene glycol solution and set to maintain a constant temperature of −3°C. Following 2 hours of exposure, flies were allowed to recover at 25°C and the proportion of individuals surviving after 24 hours was recorded. Flies of all treatment groups were assayed on the same day every time the experiment was replicated (overall three replicates, n = 11–25 flies of each sex for each diet, total n = 173 flies). In both assays, temperature was carefully monitored and adjusted throughout the experiments using calibrated thermometers placed inside one of the double jacketed chambers or in a sealed tube suspended in the water bath.

### Statistical analysis

The effect of diet composition on larval rearing efficiency (number of pupae produced by 1 g of diet), adult emergence rate and percent flyers was inferred using Kruskal–Wallis non-parametric tests. Where a significant effect was detected, a-priory pairwise comparisons were performed against a control group (8% dietary yeast, as regularly used for mass-rearing) using Wilcoxson’s test. The effect of diet on pupal weight was examined on pooled data from the three replicates using Tukey HSD comparisons. Full factorial ANOVA was used to examine the effects of diet composition and sex on lipid and protein readings after pooling data from the three replicates of each treatment group. Within the model, means were separated by Tukey HSD comparisons.

Heat and cold stress: Analyses were performed on pooled data from the three replicates. Parametric survival analysis was used to examine the effects of larval diet composition and sex on survival times of flies under heat stress. Preliminary analysis revealed that neither sex (*Χ^2^ *= 0.46, df = 1, *P* = 0.495) nor the interaction between sex and diet (*Χ^2^ *= 2.81, df = 3, *P* = 0.421) was significant predictors of survival time. Consequently, the effect of larval diet was analysed on pooled data from males and females. A generalized regression model was used to separate group means using multiple Tukey HSD comparisons. These models were fitted with a log-normal distribution function for the response variable. Regarding cold stress, diet-dependent differences in survival ratios following acute exposure to chilling were established by contingency analysis on pooled data from males and females.

As diet significantly affected adult body weight (see results), we could not include weight as an explanatory variable in these analyses. Instead, we examined the effect of weight on survival following heat and cold stress separately for males and females in each of the diet groups. For flies undergoing heat stress we examined the correlation between weight and survival time. For flies assayed for cold resistance the effect of weight on survival was examined by direct comparisons of the weight of surviving and dead flies (ANOVA). These tests indicated that in all diet groups, and regardless of thermal stress (heat or cold), weight did not significantly affect survival (heat stress: linear regression: 0.002 ≤ *F* ≤ 2.6, 0.96 ≥ *P* ≥ 0.14; cold stress: logistic regression: 0.0004 ≤ *Χ^2^* ≤ 1.64, 0.98 ≥ *P* ≥ 0.19, ANOVA: 0.0004 ≤ *F* ≤ 1.54, 0.95 ≥ P ≥ 0.22, see supplementary results, Figs. S1 and S2).

All analyses were performed using the *JMP* statistical package (SAS, Cary, NC, USA). Throughout the text, means and standard errors are reported.

## Results

### Larval performance assays

**Figure 1.**
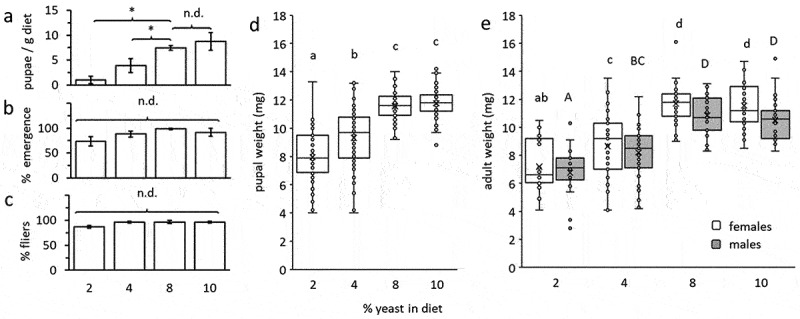
Larval performance was affected by the yeast content in the diet. (a) number of insects produced per weight of diet, (b) adult emergence rate, and (c) percent flying adults (Kruskal-Wallis and Wilcoxon tests, n = 3 replicates in each group, error bars depict the standard error, n.d. no differences, * significantly different). (d) box plots depicting pupal weight according to treatment (Tukey HSD comparisons, *P* ≤ 0.0 n = 60 individuals in each group). (e) fresh weight of 10 day old males and females participating in thermal tolerance assays, as affected by the yeast content in larval diets (ANOVA followed by Tukey HSD comparisons: *P* < 0.04, n = 21–35 individuals in each group). The number and weight of insects produced were significantly affected by yeast content in the diet. Different letters stand for significant differences between groups (small letterhead for females, capital letters for males).

The number of pupae produced per g of diet was significantly affected by the proportion of yeast in the diet (Kruskal–Wallis test: *Χ^2^* = 8.9; d.f. = 3; *P* = 0.03, Figure 1a). Diets containing 2% and 4% yeast produced significantly fewer pupae compared to diets with 8% yeast (Wilcoxon test: *Χ^2^* = 3.8; d.f. = 1; *P* = 0.04). No significant differences were detected at 8% and 10% yeast in the diet composition (Wilcoxon test: *Χ^2^* = 0.04; d.f. = 1; *P* = 0.82). Larval diets with 8% and 10% yeast produced on average 7.2–8.5 times more pupae per gram than the diet containing 2% yeast, and 1.9–2.3 times more than the diet containing 4% yeast. Nevertheless, no significant differences were detected among the proportions of emerging and flying adults derived from the four diets (Figures 1b and 1c, respectively; *Χ^2^_emergence_* = 4.25; d.f. = 3; *P* = 0.23, *Χ^2^_flyers_* = 5.57; d.f. = 3; *P* = 0.13). Pupal weight was significantly affected by the yeast content of the larval diet Tukey HSD comparisons, *P* < 0.0001 (Figure 1d). Pupae derived from diets containing 8% and 10% yeast were similar in weight, and 1.24–1.26 times (2.25–2.47 mg) heavier than counterparts reared on 4% yeast, and 1.45–1.48 times (3.61–3.82 mg) heavier than pupae derived from diets containing 2% yeast (Figure 1d). Similarly, adults derived from the different larval diets differed in their fresh weight at 10 days after emerging, and maintained in only sugar and water, before their exposure to thermal stress (Figure 1e). Adult flies (males and females) derived from 8% to 10% yeast were almost twice the weight of adults derived from 2% yeast diets (Figure 1e). Flies derived from 4% diets had an intermediate weight to flies derived from the other diets (Figure 1e).

**Figure 2.**
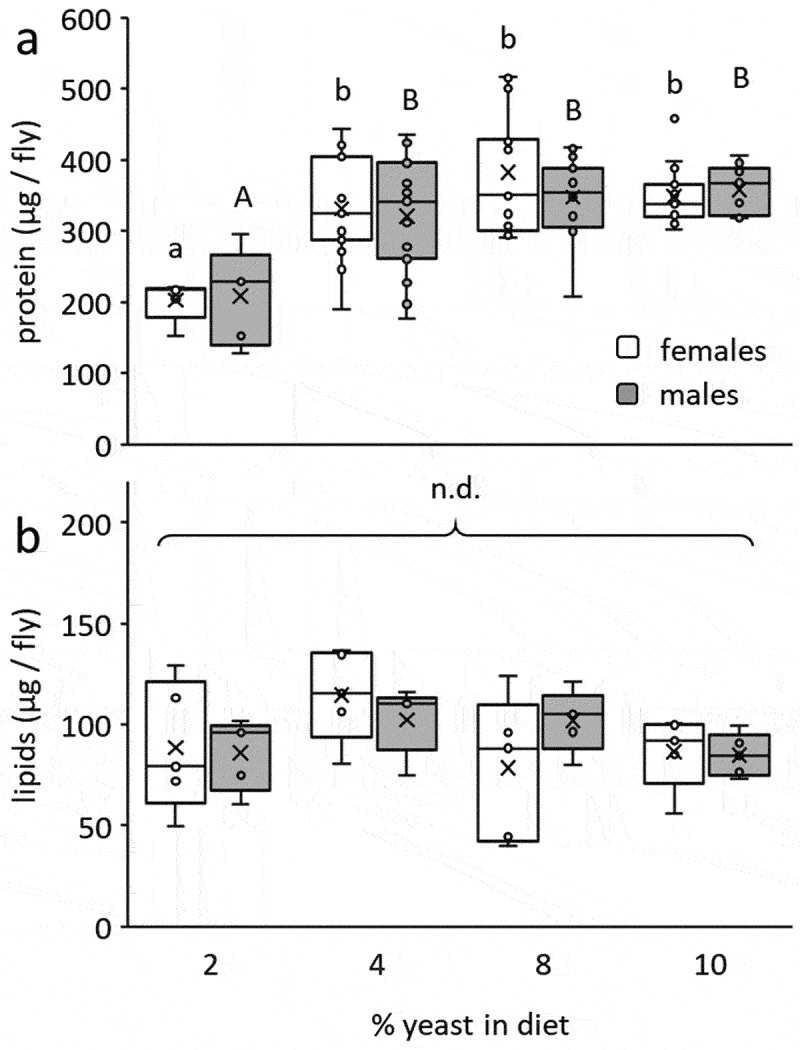
Individual protein (a) and lipid (b) content in emerging *B. zonata* males and females as affected by the yeast content in larval diets. Protein content significantly depended on diet composition (ANOVA followed by Tukey HSD comparisons: *P* < 0.017) but not sex (*P* = 0.59, n = 5–15 individuals in each group). No significant differences in lipid reserves were detected among treatment groups (ANOVA followed by Tukey HSD comparisons: *P* > 0.05) or between the two sexes (*P* = 0.81, n = 5 individuals in each group). Different letters stand for significant differences between groups (small letterhead for females and capital letters for males).

Accumulation of protein by developing insects, measured in the emerging adult, was significantly affected by the yeast content of the larval diet (*F* = 17.5; d.f. = 3, 99; *P* < 0.0001) but not by sex (*F* = 0.28; d.f. = 1, 99; *P* = 0.59) or the interaction of diet and sex (*F* = 0.67; d.f. = 1, 99; *P* = 0.56, Figure 2a). Adults developing as larvae on yeast-poor diets (2% yeast) contained significantly less protein compared to adults derived from the other three diets (4%, 8% and 10% yeast, Tukey HSD comparisons, P ≤ 0.017, Figure 2a). Contrarily, the lipid contents of adults were similar regardless of the yeast content in the diet (*F* = 2.16; d.f. = 3, 39; *P* = 0.11), sex (*F_sex_* = 0.05; d.f. = 1, 39; *P* = 0.81) or the interaction between diet and sex (*F_sex_* = 1.15; d.f. = 3, 39; *P* = 0.34, Figure 2b). Accordingly, Tuckey HSD comparisons did not separate between the groups.

### Resistance to extreme temperature conditions

**Figure 3.**
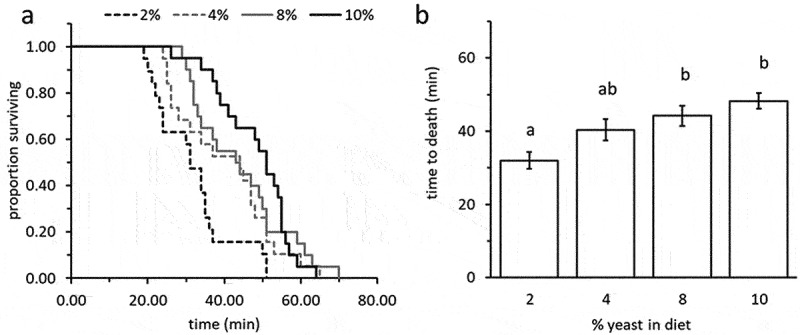
Heat tolerance (time to death) of 10 days adult *B. zonata* exposed to 42°C as affected by the yeast content in the larval diet. (a) survival plots depicting time to death (minutes) of flies in different treatment groups. (b) Survival times (means ± SE) depending on treatment. Adults developing as larvae on a protein poor, 2% yeast diet were significantly less resistant to heat stress (generalized regression followed by Tukey HSD comparisons, *t* ≤ −3.71, *P* ≤ 0.002, n = 19–20 individuals in each group). Different letters stand for significant differences between groups.

The proportion of adults surviving acute cold stress (−3°C for 2 hrs.) was similar in all treatment groups (ranged from 20% to 52%) and was not affected by the yeast content in the diet (Likelihood ratio test: *Χ^2^*_females_ = 0.45; d.f. = 3; *P* = 0.93; *Χ^2^*_males_ = 7.45; d.f. = 3; *P* = 0.06, n = 11–25 flies in each group). Nevertheless, larval diet was a significant predictor of adult response to heat-stress (42°C) (Wald test: *Χ^2^ *= 25.73, df = 3, *P* < 0.0001). Adults developing as larvae on diets containing 2% yeast died significantly faster when exposed to 42°C compared to counterparts derived from diets containing 8% or 10% yeast (12.2 and 16.2 minutes faster, respectively; generalized regression followed by Tukey HSD comparisons, *t* ≤ −3.71, *P* ≤ 0.002). Flies obtained from 4% yeast diets showed intermediate levels of heat tolerance (Figures 3a and 3b, n = 19–20 individuals in each group).

## Discussion

### Dietary yeast content and larval performance

Two main components of the larval diet were manipulated in this study: brewer’s yeast and wheat bran. The chemical composition of the brewer’s yeast usually used in our laboratory includes approximately 50% protein, 31.5% carbohydrates and 6% fats, in addition to 6–8% nucleotides and vitamins [26]. The relatively high content of protein in the brewer’s yeast has led to many authors to ‘synonymize’ changes in larval diet brewer’s yeast as modifications in protein content [see for example 20]. In the case of this study, a 2% yeast in the larval diet will constitute a 1% protein content, while a 4% yeast a 2% protein, an 8% yeast a 4% protein and a 10% yeast a 5% protein content (i.e. a five folds difference in protein content). Regarding carbohydrates (ca. 31%), 2% yeast will contribute with 0.62 g/100 g of diet of carbohydrates, while a 10% yeast will contribute with 3.1 g of carbohydrates, besides the 12 g of sucrose added to all larval diets (i.e. an extra 25% carbohydrate contents in the highest yeast concentration diet). Coarse wheat bran may also contribute to the nutritional contents of the larval diets, but its nutritional addition may be minimal. While wheat bran is known to contain proteins (ca. 15%) and carbohydrates (mainly starch), their nutritional availability is low due to their poor digestibility: proteins are embedded within a polysaccharide-matrix [27]. Wheat bran is a by-product of wheat milling and includes mainly the starchy layers of the germ and seed epidermis [28] For protein and carbohydrates to become nutritionally available, further milling processes and fermentation are needed [27,29]. In addition, wheat bran addition was higher in the 2% yeast diet (ca. 25% more to that in 10% yeast); in this diet, results of developmental parameters used to evaluate diet performance were relatively poorer than in the other diets (Figure 1). Thus, we expect that their actual nutritional contribution was relatively low.

As expected, restricting the contents of brewer’s yeast in the larval diet, and therefore of protein, as well as vitamins and other essential nutrients and minerals, negatively affected the development of *B. zonata* larvae. The number of pupae produced per g of diet was significantly reduced (Figure 1a) in 2% yeast larval diets together with pupal weight (Figure 1d), protein contents of the emerging adults (Figure 2a) and adult weight at 10 days of emergence (1e). The effect of diet on pupal production and weight is more noticeable if we consider the relatively high density of seeded eggs per g of substrate, highlighting the importance of yeast in the larval diet. Nevertheless, the viability of the pupae produced and the flight capability of the emerging adults were not affected regardless of the diet used (Figures 1b and 1c).

Protein is an essential macronutrient for developing larvae, and the detrimental effects of dietary protein deficiency have been amply documented for tephritids and other holometabolous insects [4]. Similar to our results, Nestel & Nemni-Lavi [26],reported a reduced recovery of *C. capitata* pupae, with lower pupal weight in diets with yeast content less than 4%. Additionally, Chang [30],found that the lack of essential amino acids in the diet negatively affected development of *C. capitata* larvae. Pascacio-Villafán and colleagues [31,and 20] also provided evidences of the detrimental effects of low protein in larval diets on many developmental parameters of the Mexican fruit fly, *Anastrepha ludens*. Hence, it is plausible to argue that reducing the yeast content of the diet resulted in protein deficiency for the larvae, which subsequently lead to reduction of the developmental success and reduced body size of the adults. In addition to protein, vitamins derived from yeast are also expected to contribute to larval development and were probably deficient in diets where yeast have been reduced [32].

Restricting the yeast content in the larval diet additionally reduced the protein contents in emerging adults, most probably due to lower protein reserves accumulated during the larval stage [4]. Similarly to our results, a reduced carry-over of protein reserves from larvae developing on yeast-restricted diets to emerging adults was recorded for *C. capitata* [33,34,26] and *C. cosyra* [6]. Contrary to protein, the lipid contents of emerging adults were unaffected by the yeast content of the larval diet. A similar result is reported for *C. cosyra*, where no effect of the yeast content in the larval diet was recorded on lipid reserves at adult emergence [6], and in *C. capitata* reared on different larval diets [23]. The marginal effect of yeast availability during the larval stage on lipid content of emerging adults in several tephritid flies possibly points to a more universal mechanism of lipid regulation and optimization, and suggests a compensatory metabolic mechanism active in the pupae during metamorphosis. Indeed, in *C. capitata*, the large variability in the amount of lipids accumulated by larvae reared on diets which drastically differ in their nutritional content tend to be regulated towards a similar optimal level during metamorphosis [23].

*Nutrition and resistance to extreme temperature conditions*:

The ability of the PFF to tolerate acute heat stress was significantly reduced when the yeast content in the larval diet was decreased (Figures 3a and 3b), but survival following cold exposure was not affected (although a marginal non-significant effect at the 6% probability of type 1 error was observed in males, but not in females). The observed lack of effect on cold resistance contrasts with previous studies in *D. melanogaster*, where larval nutrition affected recovery time of surviving male and female flies reared on different larval diets [10]. The lack of observed effects in our case may be related to the differences in measurements. Andersen et al. [10], measured the lapse-time required for chilled flies to recover, while we measured survival during the first 24 hrs. after chilling. This difference in methodology may have masked actual effects of larval diet on chilling resistance. This aspect requires further investigation, more in-view of the marginal effect of larval diet on PFF males’ chilling resistance.

Fresh weight was found in our study to have no effect on PFF thermal tolerance. Due to the constraints of analysing the diet-dependent fresh weights effects on binomial results (recovering vs. non-recovering) we approached the inquiry on these effects by separately analysing the effect of weight on survival in adults derived from the different larval diets (by contrasting weights of recovered vs. unrecovered flies). Our results suggested that the variability of fresh weight within each group of flies does not explain recovery from chilling (Fig. S2). Similarly, no significant relation was found between fresh weight and time to lose neuro-muscular coordination under heat stress in the individual larval diets (Fig. S1). Weight and size of flies are expected to affect resistance to thermal stress. Previous studies have demonstrated that organismal size affected resistance to stress. For instance, smaller and leaner *D. melanogaster* are less tolerant to heat stress than heavier flies [36]. While we did not detect significant effects of within-diet weight variability on the resistance of PFF to thermal stress, the fact that smaller flies derived from a 2% larval diet (Figure 1e) were less tolerant to heat stress (Figure 3), and marginally to chilling, suggest that size may in fact affect PFF tolerance to thermal stress. This aspect requires additional research.

Nutrition and thermal tolerance in larvae was previously associated particularly in relation to cold resistance. In larvae of *Drosophila* spp., diets differing in protein and carbohydrate content significantly affect the ability of the resulting adults to resist chilling [e.g. 37,38]. Similarly, Lepidopteran larvae feeding on favourable and less adequate host plants demonstrated significantly different tolerance to cold stress [e.g. 39, 40]. In some cases (i.e. *D. melanogaster*) cold tolerance was directly linked with certain nutrients in the diet of larvae [e.g. proline; 41] and adults [e.g. NaCl and KCl; 42], or nutrients mediated by gut bacteria of tephritid flies [*B. dorsalis*: arginine and proline; 43]. The above nutritional metabolites are assumed to affect the freezing temperature and ion regulation in the haemolymph, and act as protein and membrane stabilizers, thus preventing cellular damage during cold stress [41,42, and references therein]. The amount and composition of lipids gained by larvae during development may additionally affect the cold tolerance of adults [e.g. cholesterol, 44]. Using the PFF and a simple experimental setup in our study, we were unable to demonstrate any association between larval diet and adult cold tolerance, although the observed marginal effect on males’ recovery from chilling suggests that larval diet may in fact affect the PFF tolerance to chilling. Hence, more elaborated studies focusing on the mechanistic aspects of the nutrition – cold tolerance associations should be conducted.

### Larval nutrition and heat tolerance

The effect of larval nutrition on the tolerance of adults to heat has been addressed less frequently. In *D. melanogaster*, excess protein in the larval diet promoted adult tolerance to heat and desiccation stress compared to flies derived from carbohydrate-enriched larval diets [10]. Similarly, adults reared on nutritionally rich synthetic larval diets were more resistant to heat-stress compared to smaller adults derived from decomposing fruit [35, 36]. Such studies suggest that larval nutrition and protein availability may be important in determining the heat tolerance of adult insects. Nevertheless, the specific nutrients and mechanisms involved remain unclear. Possibly, low protein reserves predispose adult flies to weaken their cuticle barriers and metabolic ability to withstand dehydration during heat stress. While not clear at the present time, these changes may be mediated by protein metabolism. One possible explanation could be related to the availability of the amino-acids tyrosine and phenylalanine – serving as precursors for synthesis of melanin, which in turn performs a critical function in cuticle hardening and desiccation-resistance in insects [45]. Thus, in the case of PFF the lower yeast availability and possible deficiency in specific amino-acids during larval development may have affected the ability of adults to regulate water loss through the cuticle, and to tolerate heat-stress. Lipids may additionally indirectly contribute to heat resistance by affecting the deposition of cuticle-waxes and reducing evaporative water loss at high temperatures [46]. Another possibility is that the production of heat-shock proteins, which may provide a higher tolerance to heat-stress [47], is affected by protein restriction during larval development. Andersen et al. [10],found that Hsp70 genes of *D. melanogaster* are upregulated in larvae fed an enriched protein larval diet, and a similar scenario may be pertinent to our experiments. Nevertheless, our results are currently unable to lend support to either of these explanations.

Larval nutrition was additionally examined in relation to other environmental stressors affecting adult fruit flies such as starvation and desiccation, and in some cases these measures were examined in relation to heat stress. In *D. melanogaster* leaner and smaller adults developing as larvae in fruit were more resistant to starvation and desiccation when contrasted with flies derived from protein-rich synthetic larval diets [36]. However, these flies were less resistant to heat-stress [36], suggesting that alike cold tolerance, heat resistance and starvation resistance may be negatively associated. Studies on tephritids indicate similar associations between larval nutrition and adult starvation – desiccation resistance. Weldon et al. [6], observed that *C. cosyra* reared on protein-poor larval diets were more resistant to starvation and desiccation at adult eclosion compared to counterparts derived from protein-enriched larval diets. Such flies had significantly reduced body mass and protein contents, but maintain similar lipid reserves as adults reared on a protein-rich larval diet [6]. Pascacio-Villafán [31] reported similar effects on the Mexican fruit fly, *A. ludens*: carbohydrate-enriched larval media resulted in more tolerant newly-emerged adults to desiccation and starvation stress. Similar phenotypes in Drosophila have been associated with adult lipid reserves [48, see also 37], highlighting the function of lipids as energetic reserves and as a source of metabolic water during starvation and low humidity conditions [49, see also 6]. Nevertheless, the thermal tolerance of the flies was not examined by these studies.

To the best of our knowledge, our study is the first reporting the effect of larval nutrition on heat and cold tolerance of adult tephritids, and our results coincide with those found in *D. melanogaster*, at least as far as heat-stress tolerance is regarded [10,36]. Considering the available data on the association between larval nutrition, adult starvation and desiccation resistance and adult thermal tolerance, we assume that yeast-poor larval diets produce small adults which are relatively resistant to starvation but cope less successfully with heat stress compared to counterparts reared as larvae on yeast rich diets. The contrasting effects of nutrition, and possible trade-off between heat tolerance and dehydration – starvation tolerance point at complex physiological constraints affecting the ecology and geographic distribution of a species.

Understanding the factors affecting insect thermal tolerance is imperative for predicting climatic limits to the potential distribution of a species. Here, we include larval nutrition as an additional factor affecting thermal resistance in tephritid flies. It is important to have in mind that insect used in this experiment derived from a laboratory colony, which has been acclimated for several generation to the rearing conditions prevailing in the laboratory. Studies investigating the effects of different acclimation on heat and cold tolerance, similar to those conducted by 18, as well as investigating the genetic expression induced by heat and cold stress, could provide a deeper understanding on the mechanisms of stress tolerance involved in this and other species of Tephritidae. We highlight this aspect in light of the PFF’s current expansion through the east Mediterranean and North Africa, and the consequent agricultural threat to Mediterranean and continental Europe.

## Supplementary Material

Supplemental Material

## Data location

Provided as a supplementary file
