# Supplementary figures and images for: Larval nutritional-stress and tolerance to extreme temperatures in the peach fruit fly, Bactrocera zonata (Diptera: Tephritidae)
Source: Fly (Austin). 2022 Dec 28;17(1):2157161. doi: 10.1080/19336934.2022.2157161 (PMC9809946; doi:10.1080/19336934.2022.2157161)

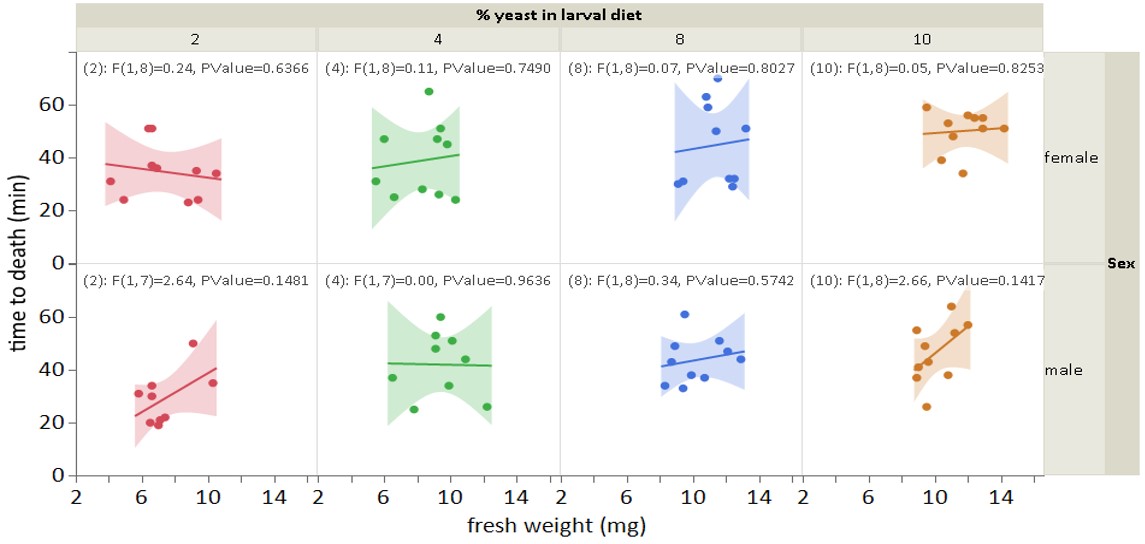

Supplement: Supplemental Material [file KFLY_A_2157161_SM9494.zip › supplementary/FigS1.jpg]

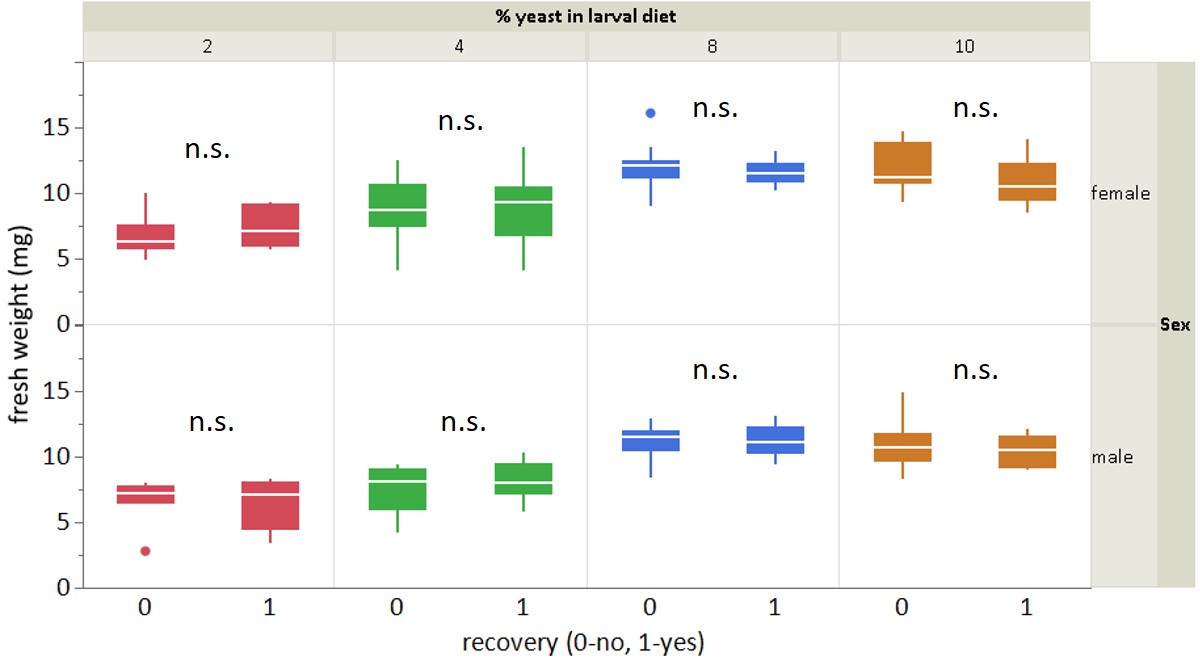

Supplement: Supplemental Material [file KFLY_A_2157161_SM9494.zip › supplementary/FigS2.jpg]
